# Interleukins in Thyroid Cancer: From Basic Researches to Applications in Clinical Practice

**DOI:** 10.3389/fimmu.2020.01124

**Published:** 2020-06-12

**Authors:** Chuang Xi, Guo-Qiang Zhang, Zhen-Kui Sun, Hong-Jun Song, Chen-Tian Shen, Xiao-Yue Chen, Jian-Wen Sun, Zhong-Ling Qiu, Quan-Yong Luo

**Affiliations:** Department of Nuclear Medicine, Shanghai Jiao Tong University Affiliated Sixth People's Hospital, Shanghai, China

**Keywords:** interleukins, thyroid cancer, inflammation, tumor microenvironment, immunotherapy

## Abstract

Inflammation is crucial to tumorigenesis and progression of many cancers. Inflammatory molecules in tumor microenvironment exert pro- or anti-tumor effects. Among them, interleukin, mainly produced by CD3+ and CD4+ T lymphocytes, is a class of small molecule proteins which play an important role in intercellular communication. Numerous studies have confirmed that interleukins are closely related to thyroid cancer. Interleukins regulate the proliferation and migration of thyroid cancer cells and they have prospects in discriminating benign and malignant thyroid diseases, predicting the risk of tumorigenesis, evaluating the prognosis and monitoring the recurrence of thyroid cancer. Besides, the effective application of interleukins in treatment of thyroid cancer has been confirmed by some cell and animal researches. The present review will introduce the potential mechanisms of interleukins in thyroid cancer and focus on the applications of interleukins in clinical practice of thyroid cancer, which will help update understanding of the progress of interleukins researches in thyroid cancer.

## Introduction

Thyroid cancer is the most common endocrine malignancy with increasing incidence rate over the past decades (1). It happens as a result of hereditary susceptibility and environment factors such as iodine excess, radiation exposure, obesity (2). According to pathology types, thyroid cancer is divided into differentiated thyroid carcinoma (DTC), anaplastic thyroid carcinoma (ATC), and medullary thyroid carcinoma (MTC). DTC, including papillary thyroid carcinoma (PTC) and follicular thyroid carcinoma (FTC), accounts for 90% of all thyroid cancers and has a relatively good prognosis. More than 80% of patients with DTC can achieve excellent response to current treatment model, such as surgery, radioiodine (RAI) therapy, and TSH suppressive therapy (3). Although ATC only accounts for 1–2% of thyroid cancer, it is responsible for 14–50% of all thyroid cancer-related deaths due to the lack of effective treatment (4). Molecular targeted therapy is the most promising emerging treatment for ATC and the involved drugs are multiple receptor tyrosine kinase inhibitors (5). MTC, derived from thyroid C cells, accounts for about 5–10% of thyroid cancer and the current treatment of MTC is limited to surgery (6).

Tumor microenvironment is closely related to the occurrence and development of cancer. It consists of immune cells, stroma cells, cytokines, and chemokines, which exert pro- or anti-tumor effects. Interleukins are small protein signaling molecules that belong to the superfamily of cytokines and are mainly produced by T lymphocytes, monocytes, macrophages, and endothelial cells. The main functions of interleukins include the facilitation of communication between cells of the immune system, regulation of transcription factors, and control of inflammation. The role of interleukins in cancer was first described by Vose (7), and in the following decades many studies have confirmed that interleukins, from IL-1 to IL-38, play significant roles in many cancers, such as breast cancer, hepatoma, thyroid cancer etc. (8, 9).

To date, numerous studies have confirmed that interleukins play significant roles in the diagnosis and treatment of thyroid cancer. This review will present the effects of interleukins in thyroid cancer and the clinical applications in the diagnosis and treatment of thyroid cancer in order to help update understanding of the progress of interleukins researches in thyroid cancer.

## The Effects of Interleukins in Thyroid Cancer

**Figure 1:**
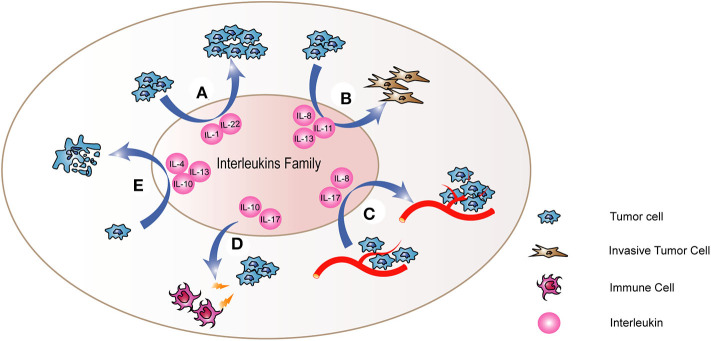
The mechanism of interleukins in thyroid cancer. Interleukins **(A)** regulate the proliferation of thyroid cancer cells and promote the process of **(B)** Epithelial-to-Mesenchymal Transition (EMT) and **(C)** angiogenesis. Besides, they also regulate the abilities of thyroid cancer cells to **(D)** resist to cell apoptosis and **(E)** escape the immune system. Through these mechanisms, interleukins cloud play important roles in the tumorigenesis and development of thyroid cancer.

Growing evidence suggests that imbalance of pro-inflammatory and anti-inflammatory cytokines is correlated to the pathogenesis of thyroid cancer. Inflammatory molecules in tumor microenvironment exert two main effects. One hand, they sustain features of the malignant phenotype of tumors, such as proliferation and invasiveness (10). Moreover, they recruit inflammatory and immune cells, and induce the remodeling of the tumor stroma and stimulate angiogenesis. Thus, inflammatory molecules could further promote tumor progression. In addition, the recruitment of immune cells into tumor sites could result in the immune escape of cancer cells, because cancer cells could induce the secretion of molecules that suppress immune responses and the recruitment of regulatory T cells (11). Interleukins are also crucial components of microenvironment of thyroid cancer and some studies confirmed that interleukins play significant roles in thyroid cancer through some potential mechanisms (Figure 1, Table 1).

**Table 1:** The mechanisms of interleukins in thyroid cancer.

**Authors**	**Year**	**Samples**	**Sample size**	**Method**	**ILs**	**Findings**
Inokuchi et al. (14)	1995	NIM1 cell lines	—	—	IL-1α	IL-1α promotes the proliferation of NIM1 cells by stimulating Ca^2+^ influx voltage-dependent Ca^2+^ channels.
Kimura et al. (15)	1992	NPA cell lines WRO cell lines	—	—	IL-1α IL-1β	IL-1α and IL-1β inhibited NPA cells growth associated with the suppression of c-myc.
Yip et al. (16)	1995	TPC-1 and NPA	—	—	IL-1β	IL-1β inhibit the proliferation and invasiveness of TPC-1 and NPA.
Ohta et al. (17).	1996	PTC cell lines: BHP and NP ATC cell lines: ARO	—	—	IL-1β	IL-1β inhibit growth of BHP and NP cells but not ARO cells.
Lin et al. (18)	1998	CGTH W-1	—	—	IL-1β	IL-1β inhibit the proliferation of follicular cell line.
Mei et al. (20)	2016	Thyroid samples Cell lines	—	—	IL-22	IL-22 induces miR-595 expression which in turn downregulate Sox17 expression and then results in increasing migration and invasion of PTC cells.
Visciano et al. (23)	2015	Thyroid samples Cell lines	PTC: 30	Cell culture, western blotting IHC	IL-8	1. IL-8 is required for EMT in thyroid cancer through IL-8–Akt–Slug pathway.2. IL-8 increases the stem features of thyroid cancer cells.
Zhong et al. (26).	2016	Thyroid samples Cell lines	ATC: 76 PTC: 100	IHCELISAPCR	IL-11	Hypoxia-induced stimulation of IL-11 production results in ATC cell invasion, migration, and EMT via the PI3K/Akt/GSK3β pathway.
Chong et al. (30)	2019	Thyroid sample Cell lines	PTC: 65 FTC: 50 ATC: 22	IHC PCR Western blot	IL13Rα2	IL13RA2 is differentially regulated in PTC and is involved in cell migration via enhancing EMT.
Gu (31)	2018	TPC-1 and ARO cell lines	PTC: 40	IHCCell culture	IL13Rα2	Knockdown of IL13Rα2 could decline the number of invading cells in PTC.
Bauerle et al. (33)	2014	Cell lines Thyroid samples Mouse model	PTC: 18	PCR	IL-8	IL-8 is an important downstream mediator of NF-kB signaling in advanced thyroid cancer growth and progression.
Carvalho et al. (36)	2017	Thyroid samples	PTC: 61, FTC:19, MTC:8	IHC	IL-17	1. Expression of IL-17 proteins in DTC and MTC tissues is higher.2. High IL-17 expression was associated with recurrence and mortality in thyroid neoplasm.
Han et al. (41)	2019	Thyroid samples Cell lines	PTC: 89 PTC+HT: 49	PCR IHC	IL-17A	1. IL-17A induced MHC class I expression and promotes inhibition of tumor immune escape in PTC with HT.2. The immune escape suppressed by IL-17A may be linked to PD-1/PD-L1 pathway.
Vella et al. (43)	2004	Cell lines	—	—	IL-4 IL-10	IL4 and IL-10 plays significantly role in protecting thyroid cancer cells from apoptosis when complicated with Graves' disease.
Stassi et al. (44)	2003	Thyroid samples	PTC: 8 FTC: 8 UTC: 5	Immunostaining Western Blotting	IL-4 IL-10	IL-4 and IL-10 protect thyroid cancer cells from cytotoxic effect of antineoplastic drugs by induce the expression of Bcl-xL and Bcl-2.
Todaro et al. (46)	2006	Thyroid samples	PTC: 8 FTC: 8 UTC: 4	Immunostaining Western Blotting	IL-4 IL-10	Autocrine of IL-4 and IL-10 in thyroid cancer results in resistance to CD95-mediated apoptosis.
Gu (31)	2018	Thyroid samples PTC cell lines	PTC: 45	PCR Flow cytometry	IL13Rα2	1. IL13Rα2 is contributed to the tumorigenesis, cell progression and invasion of thyroid cancer.2. IL13Rα2 may function as an oncogene during PTC carcinogenesis.

*ILs, interleukins; TC, thyroid cancer; DTC, differentiated thyroid cancer; PTC, papillary thyroid cancer; FTC, follicular thyroid cancer; ATC, anaplastic thyroid cancer; MTC, Medullary thyroid cancer; PCR, Polymerase Chain Reaction; ELISA, enzyme-linked immunosorbent assay; IHC, immunohistochemistry; HT, Hashimoto's thyroiditis; EMT, epithelial-to-Mesenchymal Transition*.

## Regulating Tumor Cell Proliferation

Tumor cell proliferation is an important step in tumor development. Several studies have demonstrated that interleukins could regulate the proliferation of thyroid cancer cells.

IL-1 includes two activator cytokines IL-1α and IL-1β, as well as an inhibitory cytokine, the IL-1 receptor antagonist (IL-1ra). IL-1α and IL-1β bind to the same receptor, the type 1 IL-1 receptor (IL-1R), and activate the downstream signaling cascades, ultimately promoting the immune and inflammatory responses (12). The role of IL-1 in cancer has been well-demonstrated (13) and it is well-demonstrated that IL-1 could regulate the proliferation of thyroid cancer through different mechanisms. Due to different thyroid cancer cell lines used in different studies, the results are contradictory. IL-1α could promote the proliferation of PTC cell line NIM1 via stimulation of Ca^2+^ channels (14). IL-1 could also suppress the proliferation of thyroid cancer cells. IL-1 inhibits the growth of the thyroid cancer cell line NPA, which was in part associated with the suppression of c-myc (15). IL-1β exerts strong antitumor effects on PTC (16, 17) and FTC cell lines (18) through suppressing proliferation and invasiveness. Furthermore, IL-1β did not have an anti-proliferative effect on ATC cell lines, which indicates that PTC cancer cells escaping from antitumor effect of IL-1β may be a step toward anaplasia change, resulting in more aggressiveness of thyroid cancer (17). However, the mechanisms of this process are not clear and further studies are needed.

IL-22, produced by Th17 and Th22 cells, exerts its biological effects through binding to IL-22 receptor and IL-10 receptor. IL-22 triggers a variety of downstream signaling pathways including JAK/STAT3 and MAPK, resulting in cancer progression (19). In thyroid cancer, IL-22 induces miR-595 expression, which in turn downregulates Sox17 expression, thereby enhancing the migration and invasion of thyroid cancer (20).

## Promoting Epithelial-to-Mesenchymal Transition (EMT)

EMT is a process in which epithelial cells lose adhesion properties and turn into a mesenchymal phenotype, allowing non-invasive tumor cells to attain the ability of invasion and metastasis (21). It is an essential step in successful migration and metastasis of tumor cells. Some interleukins promote the EMT process of thyroid cancer and then enhance the aggressiveness of thyroid cancer.

IL-8, a pro-inflammatory chemokine, functions through binding to CXCR1 and CXCR2. Considering the characteristic expression of CXCR1 and CXCR2 on cancer cells, endothelial cells, and tumor-associated macrophages, the increased secretion of IL-8 from tumor cells has significance to the tumor microenvironment (22). IL-8 has been repeatedly reported to be a tumor-promoting cytokine in several cancers, but rarely reported in thyroid cancer. Mast cells, which correlate to malignant features and invasiveness of thyroid cancer, are the main source of IL-8 in thyroid cancer. IL-8 is required for mast cells mediated EMT in thyroid cancer through the IL-8-Akt-Slug pathway (23).

IL-11 interactives with its receptor IL-11Rα and activates signaling pathways of targeted cells such as JAK/STAT, MAPK, Src-family kinases, and PI3K pathway (24). The IL-11 gene is a hypoxia-inducible gene whose expression is induced by hypoxia via HIF-1α (25). IL-11 promotes the invasion, migration and EMT of ATC cell via the PI3K/Akt/GSK3β pathway (26). Higher expression of IL-11 in ATC tissues than in PTC could explain the higher metastasis rates of ATC (26). The promotion of EMT induced by IL-11 could take part in this process.

IL13Rα2 is a type II cytokine receptor with high binding affinity to IL-13 (27) and it has an oncogenic role in many cancers (28, 29). In PTC, IL13Rα2-induced cell migration is associated with the upregulation of EMT markers such as N-cadherin, Vimentin and Snail, indicating that IL13Rα2 enhances thyroid cancer aggressiveness through promoting EMT process (30). A recent study found that the number of invading cells in PTC declined significantly after IL13Rα2 knockdown, indicating that IL13Rα2 is involved in the invasion of PTC cells (31). However, the potential mechanism of how IL13Rα2 influence the EMT process of thyroid cancer is not clear.

## Promoting Angiogenesis

To support the high proliferation of cancer cells, tumors need to rapidly develop a new vascular network. Angiogenesis, the formation of new blood vessels, is one of crucial steps in tumor progression. NF-κB is a key regulator of angiogenesis in thyroid cancer (32), and IL-8 may be a significant downstream effector of NF-κB signaling pathway in the progression of advanced thyroid cancer (33). IL-17 is involved in the pathogenesis of inflammatory responses and is known to induce the production of IL-1β and TNF-α in the tumor microenvironment (34). The expression of IL-17 is observed in various tumor tissues and considered as the most important pro-angiogenic mediator (35). The expression level of IL-17 is higher in DTC and MTC than that in benign thyroid neoplasms (36), which suggests that IL-17 is closely correlated to the aggressiveness of thyroid cancer, and the tumor pro-angiogenesis of IL-17 could have roles in this process. However, no study has ever reported the pro-angiogenesis of IL-17 in thyroid cancer.

## Regulating Tumor Immune Escape

Human immune system is capable of recognizing and resisting cancer cells, however, by altering the host immune system, tumors can escape immune control and continue to progress (37). The tumor microenvironment provides conditions for tumors to escape the immune surveillance, and some interleukins play an important role in this process.

IL-10 is an anti-inflammatory and immunosuppressive cytokine that influence the course of cancer by promoting immune escape through inhibition of the antitumor activity of immune cells (38). IL-10 is expressed in thyroid cancer and influence the aggressiveness of it (39). The immunosuppressive effect of IL-10 may be involved in the immune escape of thyroid cancer cells and promote the aggressiveness of thyroid cancer. However, studies are needed to explain the mechanisms of this process.

Tumor cells achieve immune escape by downregulating the expression of major histocompatibility complex (MHC) class I and loss of MHC class I expression is a frequent mechanism of tumor immune escape in PTC (40). However, after IL-17 treatment, the membrane expression of MHC class I in K1 and PTC-1 increased significantly (41). Programmed cell death ligand 1 (PD-L1), expressed on the surface of tumor cells, binds to its receptor PD-1 on T cells membrane, inducing T cells anergy. The PD-1 expression of T cells reduced in the presence of IL-17 (41). It is suggested that IL-17 inhibit tumor immune escape by upregulating MHC class I expression on tumor cells and suppressing PD-L1/PD-1 pathway.

Some interleukins can inhibit the anti-tumor immune response, allowing tumor cells to escape recognition and attack by the immune system. Therefore, tumor cells can proliferate and metastasize to distant organs. Better understanding of the mechanisms of interleukins in immune escape will provide new targets for immunotherapy of thyroid cancer.

## Inhibiting Cancer Cell Apoptosis

Apoptosis, also called programmed cell death, is finely regulated at the gene level to orderly remove damaged cells (42). Its alteration is not only responsible for tumor progression but also for tumor resistance to therapies.

Autocrine production of interleukins in thyroid cancer results in upregulation of anti-apoptotic proteins, which contributes to tumor progression. IL-4 is a pleiotropic cytokine produced by Th2 cells and exerts regulatory effect on the immune response. It can induce Th2 cell proliferation and differentiation, and inhibit apoptosis of B and T cells. A variety of malignancies, such as melanoma and breast cancer, express IL-4 receptor (IL-4Rα), and IL-4 has antiproliferative and/or proapoptotic effects in these cancer cells. On the contrary, IL-4 weakly stimulates the proliferation of thyroid cancer and protects it from apoptosis. The pro-tumor effect of IL-4 is associated with the up-regulation of anti-apoptotic molecule Bcl-2 and the weak down-regulation of the pro-apoptotic molecule Bax (43). Besides, autocrine production of IL-4 and IL-10 induces the over-expression of Bcl-xL and Bcl-2, two anti-apoptotic proteins, which subsequently protect thyroid cancer cells from the cytotoxic effects of antineoplastic drugs (44). Both PTC and FTC cells express CD95 and its ligand CD95L, which mediate cell apoptosis, however, expression of CD95 and CD95L does not affect tumorigenesis and progression in thyroid cancer (45). Therefore, there is a molecular mechanism that restrains the CD95-mediated apoptosis signaling pathway in thyroid cancer and autocrine production of IL-4 and IL-10 may have significant roles in this mechanism, because IL-4 and IL-10 promote resistance to CD95-mediated apoptosis via the activating the Jak/Stat pathway and up-regulating cFLIPL and PED (46). Besides, in PTC cell lines, transfection with siRNA targeting IL13Rα2 induces cell apoptosis by upregulation of caspase 3 and then results in inhibition of cell proliferation, which indicates that IL13Rα2 promotes the invasion and metastasis of tumors through inhibiting apoptosis (31).

Although the mechanisms of some interleukins in thyroid cancer have been described by some studies, how other interleukins confirmed to be correlated to thyroid cancer in clinical researches affect the progression of thyroid cancer is still not clear so far. Therefore, in order to provide more help for treatment of thyroid cancer, further studies focused on potential mechanisms in are still needed.

## Clinical Utilities of Interleukins in Thyroid Cancer

**Figure 2:**
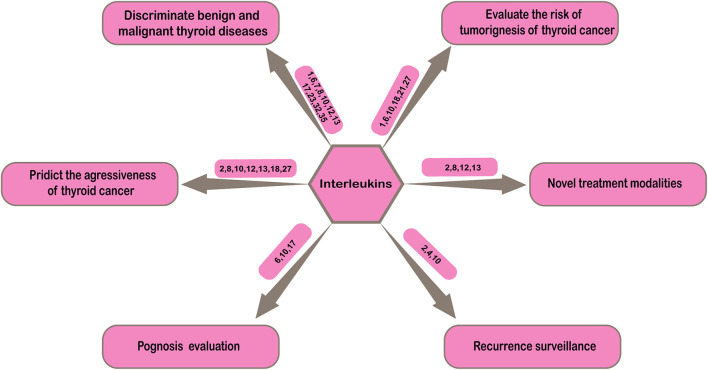
Clinical applications of interleukins in thyroid cancer.

Thyroid cancer is the most common malignant tumor of endocrine system with increasing incidence over recent decades (1). How to improve the diagnosis and treatment quality of thyroid cancer has been paid more attention by clinicians. In clinical practice, the diagnosis, treatment, prognosis evaluation, and disease recurrence monitoring of thyroid cancer are challenging. Either in process of initial diagnosis or postoperative pathological classification, discriminating benign and malignant thyroid nodules is not easy. In order to achieve the purpose of early detection, early diagnosis and early treatment, determination of the high-risk population is significant in clinical practice. Evaluation of aggressiveness of thyroid cancer can provide more information for accurate risk stratification of patients. In addition, most thyroid cancer progress slowly and patients has long survival time after treatments, thus the prognosis evaluation and follow-up after treatment are particularly important. These problems in clinical practice suggest more effective biochemical indicators are needed to make up for the imperfections of current clinical diagnosis and treatment of thyroid cancer in order to improve the level of clinical diagnosis and treatment and make patients attain more benefits. This section will concentrate on the possible applications of interleukins in the clinical practice of thyroid cancer in order to provide more help for clinicians (Figure 2).

## Discriminating Benign and Malignant Thyroid Diseases

### Serum Interleukin Level

Serum biomarkers are generally used for tumor screening due to their effectiveness and convenience, and specific serum tumor biomarkers are important for tumor diagnosis. With the development of tumor immunity, some studies confirm the possibility of serum cytokine level for tumor diagnosis. It has been reported that the serum level of some interleukins could serve as potential biomarkers in the diagnosis of thyroid cancer, especially in discriminating benign and malignant thyroid diseases (Table 2). However, the results of different studies are contradictory.

**Table 2:** Discriminate benign and malignant thyroid diseases.

**Authors**	**Year**	**Samples**	**Sample size**	**Method**	**ILs**	**Findings**
Kammoun-Krichen et al. (47).	2012	Serum samples	PTC: 15	Multiplex technology	IL-1β	Serum IL-1β levels was under expressed in PTC group compared to healthy control group and other thyroid diseases.
Niedzwiecki et al. (48)	2008	Serum samples	PTC:21 FTC:8 MTC:12 ATC: 11	ELISA	IL-1ra	Serum IL-1ra level is associated with the development of ATC and FTC.
Provatopoulou et al. (49).	2014	Serum samples	TC: 20	Multiplex technology	(IL)6, 7, 10, 13	Serum level of IL-6, IL-7, IL-10, and IL-13 was higher in thyroid disease, while IL-8 was lower than healthy controls.
Beksac et al. (50)	2016	Serum samples	PTC: 31	ELISA	IL-6, IL-8	Serum IL-6 and IL-8 were higher in presurgical thyroid samples and returned to normal following surgery.
Martins et al. (51)	2017	Serum samples	DTC: 200	ELISA	(IL)2, 6R, 8, 12	1. Serum concentration of IL-2, IL-6R, IL-8, and IL-12 might assist in the characterization of thyroid nodules' malignancy and tumor aggressiveness.2. Concentration of serum IL-8 was higher in the malignant group compared with controls.3. IL-6 and IL-10 were not able to discriminate the benign and malignant groups from controls.4. Serum concentrations of IL-2, 2R and 10 were associated with thyroglobulin levels.
Zhang et al. (52)	2018	Serum samples	PTC: 29 FTC: 11	ELISA	IL-17	Serum IL-17 level was significantly increased in patients with DTC.
Jiang et al. (53)	2014	Serum samples	TC: 11	ELISA	IL-17	Concentration of serum IL-17 was significantly higher in patients with thyroid tumors.
Lu and Yuan (54)	2015	Serum samples	PTC: 42	ELISA	IL-17	Serum IL-17 level was higher in thyroid cancer than that in thyroid adenoma.
Linkov et al. (55)	2008	Serum samples	TC: 23	Multiplex technology	IL-8 and IL-12	A panel of four serum biomarkers (IL-8, HGF, MIG, and IL-12) might assist in the discriminating thyroid cancer and benign thyroid diseases.
Basolo et al. (56)	1998	Thyroid samples	TC: 99	IHC	IL-6	Down-regulation of serum IL-6 may be a biomarker of UTC.
Ruggeri et al. (57).	2002	Thyroid samples	TC: 130	IHC	IL-6	IL-6 is negatively correlated to aggressiveness of thyroid cancer.
Zhao et al. (58).	2009	Thyroid samples	PTC: 13	IHC	IL-13	PTC expressed higher IL-13 protein and PTC had more IL-13 genetic changes compared to benign nodules.
Jiang et al. (59)	2017	Thyroid samples	PTC: 60	PCR IHC	IL-17	IL-17 is correlated to tumor TNM stage, capsule invasion, and lymph node metastasis of thyroid cancer.
Carvalho et al. (36)	2017	Thyroid samples	PTC: 61 FTC: 19 MTC: 8	IHC	IL-17	1. Expression of IL-17 proteins in DTC and MTC tissues are higher than healthy controls. 2. High IL-17 expression was associated with recurrence and mortality in thyroid neoplasm.
Plantinga et al. (60)	2013	Serum samples Thyroid samples	TC: 139 HC: 138	IHC PCR	IL-32	IL-32 protein was expressed higher in thyroid cancer tissues.

*ILs, interleukins; TC, thyroid cancer; DTC, differentiated thyroid cancer; PTC, papillary thyroid cancer; FTC, follicular thyroid cancer; UTC, undifferentiated thyroid cancer; ATC, anaplastic thyroid cancer; MTC, Medullary thyroid cancer; PCR, Polymerase Chain Reaction; ELISA, enzyme-linked immunosorbent assay*.

IL-1 consists of IL-1α, IL-1β, and IL1 receptor antagonist (IL1ra). Compared with healthy controls, IL-1β was found to be underexpressed in the serum of patients with PTC, and it was considered to be a valuable factor in discriminating atrophic thyroiditis and thyroid cancer (47). IL1ra inhibits the pro-inflammatory effects of IL-1 through competitively binding to the IL-1 receptor (61). Compared with the control group, the serum level of IL1ra is higher in patients with FTC or ATC, but not in patients with PTC or MTC (48), which indicates that serum IL-1ra level could be used as a biomarker for FTC or ATC. However, this effect reaches significance only in women. It could due to the fact that production of IL-1ra in monocytes from female patients is increased (62).

IL-6 is a pleiotropic cytokine which could induce pro- and anti-inflammatory effects under specific conditions. In tumor microenvironment, IL-6 interacts with its receptor and associates with the target cell membrane glycoprotein 130, inducing pro-inflammation cytokines production to support the chronic inflammation. In addition, the IL-6 pathway could transmit positive signals to tumor cells, promoting the proliferation, invasion and anti-apoptosis of cancer. The published researches reported that IL-6 expression is higher in patients with benign and malignant thyroid neoplasms than healthy controls, and it is associated with tumor aggressiveness and poor survival (49). Besides, serum level of IL-6 is high in presurgical serum samples of PTC patients and returns to normal level following surgery (50). However, a recent study reported that IL-6 could not discriminate benign and malignant groups from healthy controls (51). This difference may due to different interleukins measurement methods and sample size.

The findings of serum IL-8 level in thyroid cancer are contradictory in different studies. Some studies found that the serum level of IL-8 is lower in thyroid disease than healthy controls (49), while others found that the level of IL-8 expression in serum samples of PTC patients is higher than healthy controls (50). Besides, a recent study (51) reported that compared with the benign thyroid disease groups, higher concentration of IL-8 in thyroid cancer was observed. The measurement methods and sample size in these studies are different, which indicates that a multicenter large sample study is needed to determine the serum expression level of IL-8 in thyroid cancer.

Serum level of IL-17 increases significantly in patients with thyroid tumors compared with healthy controls (52, 53). And its serum expression level of thyroid cancer is higher than that of thyroid adenoma, suggesting that it could be used as a potential biomarker to distinguish thyroid cancer from adenoma (54).

Serum level of other interleukins could also be used in discriminating benign and malignant thyroid diseases. Serum levels of IL-7, IL-10, and IL-13 are higher in thyroid diseases than healthy controls (49). However, a recent study (51) reported that serum concentrations of IL-10 could not discriminate benign and malignant groups from healthy controls. Besides, Serum concentration of IL-35 in thyroid cancer is lower than that in thyroid adenoma, which indicates IL-35 could be used in discriminating thyroid cancer and adenoma (54).

Interleukins are potential biomarkers in discriminating benign and malignant thyroid diseases, however, the clinical application value of single type of interleukin still have some limitations. In order to solve this problem, some researchers have begun to focus on the combined detection of different types of interleukins or the combined detection of interleukins and other biochemical indicators. It is reported that the combination of IL-13 and IL-8 is highly efficient in identify thyroid diseases (AUC 0.90) (49). Another study evidenced that a panel of four serum biomarkers (IL-8, HGF, MIG, and IL-12) might assist in discriminating thyroid cancer and benign thyroid diseases (AUC 0.81) (55).

Serum interleukin level is correlated to thyroid cancer and could be used to discriminate benign and malignant thyroid diseases. However, due to the different measurement methods and sample size, the results of different studies are not similar and even opposite. Besides, there are still some limitations in the effect of single type of interleukin. At present, few studies focused on the combined detection of different types of interleukins or the combined detection of interleukins and other biochemical indicators, which also limits the application of serum interleukins in clinical practice (49, 55). Therefore, in order to improve the clinical application value of interleukins, it is necessary to use the same detection methods for multi-center, large sample size research. In addition, studies on the combined detection are also needed.

### Expression of Interleukins in Thyroid Tissues

Effective biomarkers are needed to improve the accuracy of pathological diagnosis of thyroid cancer. Even though serum interleukins could be used to discriminate benign and malignant thyroid diseases, they might not accurately reflect their actual expression level in thyroid tissues. Thus, studies on expression level of interleukins in thyroid tissues are still needed. Previous studies showed that IL-6 expression was significantly down-regulated in undifferentiated thyroid cancer tissues (56). Besides, PTC tissues had the highest level of IL-6 expression while FTC and ATC issues were consistently negative for IL-6 expression (57). Compared with benign or normal thyroid tissues, higher expressions of IL-13 (58) in PTC tissues, higher expressions of IL-17 (59) and IL-23 (36) in DTC and MTC tissues were observed. Besides, the expression of IL-32 (60) in thyroid cancer issues is also higher in benign thyroid tissues.

The expression of interleukins in thyroid cancer tissues is significantly different compared with benign or normal thyroid tissues. Therefore, interleukins could be potential biomarkers in the pathological diagnosis of thyroid cancer, and a simple immunohistochemical analysis in thyroid tissues could help pathologists discriminate benign and malignant thyroid disease accurately. However, similar to serum interleukins, further studies on the expression of different interleukins and other biomarkers in thyroid cancer are needed to improve their clinical value.

### Evaluating the Risk of Tumorigenesis of Thyroid Cancer

Heritable factors are crucial to the occurrence and development of cancers. Several single nucleotide polymorphisms (SNPs) found in cytokine genes affect the expression or function of proteins which have been evaluated for their roles in cancer predisposition (63). Considering that genetically inherited predisposition is the initiating factor to thyroid cancer occurrence, some studies have focused on gene polymorphisms of interleukins and found some favorable results. The interleukins of whose SNPs are associated with the increased risk of PTC include IL-1α (64), IL6 (65, 66), IL10 (67, 68), IL-18 (69), and IL-27 (70). IL-1β (64, 71) and IL-21 (72) gene polymorphisms were related to the decreased risk of tumorigenesis in thyroid cancer. However, another study found that IL-27 gene polymorphism was not a risk factor of tumorigenesis but a risk factor lymph node metastasis in PTC (73). Patient selection bias may account for this difference. Moreover, the study focused on the serum interleukins has shown that high serum IL-10 level was positively associated with an increased risk of thyroid cancer, but it was significant only in women (74).

Multiple genetic studies have demonstrated the association between interleukins and the risk of thyroid cancer. Therefore, interleukin gene testing of high-risk populations, including patients with family history of thyroid cancer or radiation exposure, could help to assess the risk of thyroid cancer more accurately. Due to the differences of heritable factors among different populations, genetic studies are needed in different population to determine the clinical value of interleukins in the risk of thyroid cancer. In addition, interleukins in human body are affected by many other diseases, such as thyroiditis, immune diseases, or other malignancy. However, there are no reports on the tumor risk assessment of patients suffering from thyroid cancer combined with other diseases. Furthermore, it is unclear how these genetic polymorphisms affect the function and production of interleukins.

### Predicting the Aggressiveness of Thyroid Cancer

Clinicopathological factors such as tumor size, extrathyroid extension, lymph node metastasis, and distant metastasis are common indicators of the aggressiveness of thyroid cancer. Aggressiveness is a significant factor for tumor risk stratification in clinical practice. Thyroid cancer patients with higher aggressiveness need more active surgical resection and radioiodine treatment. However, it is not always easy to distinguish these patients from others in initial diagnosis. At present, some studies have explored the possibility of interleukins in predicting the aggressiveness of thyroid cancer and have obtained promising results.

High levels of serum IL-2, IL-10, and IL-12 are correlated to the aggressive tumor characteristics in patients with DTC (51). Another study also reported that higher levels of positive expression of IL-10 in thyroid cancer tissues were significantly correlated to extrathyroidal invasion and larger tumor size (39). Recently, high expression of IL-13RA2 was also observed to be correlated with advanced tumor stage in PTC tissues (30).

Lymph node metastasis is the most common type of thyroid cancer metastasis. Most patients are detected with multiple lymph node metastases at the time of initial diagnosis. It is reported that interleukins are significantly associated with lymph node metastasis of thyroid cancer. The expression level of IL-8 is higher in thyroid cancer tissues with lymph node metastases than that without lymph node metastases (75). Gene polymorphism evidenced that IL-8 may contribute to DTC lymph node metastasis (76), and IL-1β may cause PTC lymph node metastasis (71). However, gene polymorphism IL-18 (69) and IL-27 (73) are negatively associated with lymph node metastasis in patients with PTC.

The correlation between interleukins and aggressiveness of thyroid cancer has been confirmed, indicating that interleukins might be used to predict the aggressiveness of thyroid cancer and provide some information for clinicians to make suitable treatment decisions. Distant metastasis is the main cause of disease-specific death in thyroid cancer patients (77). None of the above studies found the correlation between interleukins and distant metastasis of thyroid cancer. Considering the small proportion of patients with distant metastasis in these studies, the results may have some limitations. Therefore, further studies should focus on the patients with distant metastasis, and determine whether interleukins can be used to predict the risk of distant metastasis.

### Prognosis Evaluation and Recurrence Surveillance

Considering the utility in predicting recurrence and mortality of thyroid cancer, postoperative risk estimation is recommended to guide radioiodine therapy and follow-up strategies (78). Molecule profile enriches the risk estimate system and is considered a prognostic factor of thyroid cancer. More and more studies reported that interleukins are associated with survival of thyroid cancer patients and are considered as potential predictors of prognosis. Elevated serum IL-6 level is significantly associated with poor overall survival in PTC patients (79). Besides, higher expression of IL-10 in cancer tissues (39) and IL-17 in serum (36) are related to shorter recurrence-free survival of thyroid cancer patients.

Although the prognosis of DTC patients is relatively favorable, the recurrence rate after initial treatment reaches up to 8–23% (80). Therefore, recurrence monitoring during follow-up is of great significance to provide timely and accurate information to patients for intensive treatment. Serum thyroglobulin (Tg) level after thyroidectomy and iodine ablation is the most effective indicator of disease recurrence during follow-up, with high sensitivity and accuracy. Interleukins such as IL-2 and IL-10 were correlated to tumor aggressiveness and to serum Tg level, which suggests the involvement of interleukins would improve the efficiency of Tg evaluation system (51). For DTC patients with autoimmune disease or post-trauma immune system response, the evaluation efficiency of Tg is greatly reduced due to interference of thyroglobulin antibody (TgAb). Thus, effective indicators are needed to monitor tumor recurrence in these patients. In PTC patients with or without Hashimoto's thyroiditis, serum IL-4 and IL-10 levels were reported to be higher in cases with persistent or recurrent disease than those without persistent or recurrent disease, suggesting that they could select patients who need close monitoring and intensive treatment (81).

Taken together, interleukins could be potential biomarkers used in prognosis evaluation and disease surveillance of thyroid cancer. The risk stratification information based on interleukin levels and clinicopathologic features could guide follow-up management decisions of patients with thyroid cancer. However, many other diseases also affect the production and function of interleukins in human body. Therefore, more studies are needed on thyroid cancer patients combined with other diseases to further confirm the potential utility of interleukins in prognosis evaluation and recurrence surveillance of thyroid cancer.

## Current Situation of Interleukins in Treatment of Thyroid Cancer

Eighty percentage of DTC patients respond well to the current combined treatment model of surgery, RAI therapy, and TSH suppression. However, ~10% of DTC patients have distant metastasis at the time of diagnosis or develop distant metastasis during follow-up, and these patients usually have poor response to treatments (3). Besides, some DTC patients gradually progress to be refractory to RAI (RR-DTC) during treatment. There is no effective treatment for aggressive pathological types of thyroid cancer, such as ATC. Therefore, new therapy model for these patients are needed. Over recent decades, it has been suggested that some interleukins have good prospects in the treatment of thyroid cancer (Table 3).

**Table 3:** Current situation of treatments targeting on interleukins in thyroid cancer.

**Authors**	**Year**	**Samples**	**ILs**	**Findings**
Zhang et al. (84)	1999	Wag/Rij rats	IL-2	1. AdCMVmIL2 has antitumor effects and could establish tumor immune in MTC animal model. 2. AdCMVmIL2 has low toxicity
Cressent. et al.(85)	1995	WagRij rats	IL-2	1. Injection of IL-2 or IL-4 inhibit the growth of tumor in MTC animal model. 2. IL-2 and IL-4 were synergistic in their inhibitory effects
Zhang and DeGroot (86)	2001	Wag/Rij rats	IL-2	1. AdCMVTKhIL2 destroyed 63% of tumors in MTC animal model. 2. The antitumor effect of AdCMVTKhIL2 is superior than each single vector
Barzon et al. (87)	2003	nude mice	IL-2	A retroviral vector expressing HSV-TK and IL-2 completely eradicate tumors of ATC and reduce more than 80% tumor size of DTC
Barzon et al. (88)	2002	nude mice	IL-2	A new targeted vector of which viral enhancer replaced by human Tg gene enhancer has antitumor effect to DTC
Vitale et al. (89)	2013	TT cells MTC patients	IL-2	Combination of IL-12 and LAN could suppress MTC cells and improve quality of life in MTC patients
Iwahashi et al. (90)	2002	TPC-1 and TT cell line	IL-8	RET induces IL-8 production from both PTC and MTC cells through many signal pathways
Broutin et al. (91)	2011	TT cell line Animal model	IL-8	Sunitinib decreases the serum level of IL-8 in mice model
Yamazaki et al. (92)	2002	Animal model	IL-12	1. AdTCPmIL-12 has antitumor effects on tumor in MTC animal model. 2. AdTCPmIL-12 induces tumor bearing rats to establish long-time tumor immune
Zhang and DeGroot (93)	2000	Animal model	IL-12	1. AdCMVmIL-12 has antitumor effects on tumor in MTC animal model. 2. AdCMVmIL-12 induced tumor bearing rats to establish long-time tumor immune
Yamazaki et al. (94)	2004	WAG/Rij rats	IL-12	The combination of AdTCPtk and AdTCPmIL-12 has stronger antitumor effects on MTC than each single vector in MTC animal model
Zhang and DeGroot (95)	2003	Rats model	IL-12	AdCMVIL-12 has antitumor effects in FTC animal model
Shi et al. (96)	2003	Nude mice	IL-12	1. A single-chain IL-12 fusion protein has antitumor effects in ATC animal model. 2. Long-time tumor immune was not observed in this study
Gu (31)	2018	TPC-1 and ARO cell line	IL13Rα2	The number of invading cells declined significantly after the knockdown of IL IL13Rα2

*ILs, interleukins; MTC, medullary thyroid cancer*.

IL-2 exerts its antitumor effect by activating cytotoxic T lymphocytes, and natural killer (NK) cells. Genetic immunotherapy of IL-2 has a promising prospect in the application of the treatment of thyroid cancer, especially for ATC and MTC (82, 83). In animal models of MTC, about 42.9% of cases were cured by directly intratumoral injection of Replication-defective adenovirus expressing IL-2 (AdCMVmIL2) and most of the cured rats developed systemic immunity (84). Several studies have evaluated the possibility of IL-2 combined with other treatments to enhance the antitumor effects. The tumor growth can be inhibited and even eliminated after injection of IL-2 or IL-4. Moreover, IL-2 and IL-4 have a synergistic inhibitory effect (85). The combination of suicide gene transfer and immunotherapy provide promising results for gene therapy for thyroid cancer. An adenoviral vector expressing thymidine kinase of herpes simplex virus (HSV-TK) and IL-2 (AdCMVTKhIL2) was constructed, and about 63% of MTC tumors were destroyed after intratumoral injection of itAdCMVTKhIL2 and the antitumor effect of AdCMVTKhIL2 is superior than each single vector (86). Besides, a retroviral vector expressing HSV-TK and IL-2 could completely eradicate ATC tumors and reduce DTC tumor size by more than 80% (87). To further optimize this therapeutic approach, new vector was constructed by replacing the viral enhancer with the enhancer sequence of the human Tg gene. This new vector allows selective transgene expression and cell killing in DTC cells but not in ATC cells (88). In addition, an *in vitro* and *vivo* study suggested that combination of IL-12 and lanreotide (LAN), a somatostatin analog, could suppress and kill MTC cells and improve quality of life of MTC patients (89).

RET mutations are responsible for the course of human cancers (90). RET stimulation with its ligand GDNF induced IL-8 production in thyroid cancer (97), In MTC animal model, the decrease of serum IL-8 level is induced by RET inhibitor Sunitinib (91). IL-8 promotes the proliferation, survival, invasion, and angiogenesis of tumor. Therefore, inhibiting activation of RETof thyroid cancer, which results in the reduction of IL-8 secretion, may be an effective strategy for thyroid cancer treatment. And the decreased expression of IL-8 could be used to evaluate the curative effect of RET-inhibited treatment. It is gradually accepted that cancer stem cells (CSC) promote tumor growth, metastasis, recurrence and drug-resistance. CSC is more abundant in ATC sample than DTC (98). In human PTC specimens, a significant correlation between Mast cell (MC) density and stemness features was observed. It is reported that MC-dependent IL-8–Akt–Slug pathway that sustains EMT/stemness of thyroid cancer cells (23). Thus, the IL-8-CXCR1/2 axis might be used as a targeted therapy in advanced thyroid cancer.

Due to its most significant antitumor activity among all cytokines, IL-12 is one of the most promising interleukins in clinical application. Both IL-12 gene therapy and recombinant protein therapy inhibit thyroid cancer growth and prolonged survival (99). However, systemic administration of recombinant IL-12 caused dose-dependent toxicity in animals. In order to alleviate this situation, some studies have investigated the local expression of the IL-12 induced by transduction of IL-12 expressing vectors into the tumor tissue. Injection of AdTCPmIL-12 (92) and AdCMVmIL-12 (93) into MTC tumors has antitumor effect to both primary and distant lesions, and long-term antitumor immune was established. The combination of AdTCPtk and AdTCPmIL-12 has stronger antitumor effect to MTC than each single vector *in vivo* study (94). In addition, AdCMVIL-12 has the same antitumor effect in FTC animal model (95). Besides, a single-chain IL-12 fusion protein has antitumor effect in ATC animal model, however lone-time tumor immune was not observed in this study (96).

IL13Rα2 could serve as a target of therapeutic intervention of some malignant tumor and current trials mainly involve glioblastoma multiforme (100). In thyroid cancer, IL13Rα2 was observed to promote aggressive behavior of tumor through promoting EMT (30). The number of invading cells declined significantly after the knockdown of IL IL13Rα2, indicating the possibility of IL13Rα2 used as a novel target in the treatment of thyroid cancer (31). However, no study has reported IL13Rα2 could be used in treatment of thyroid cancer.

Interleukins have good prospects in the treatment of thyroid cancer. Studies mentioned above have reported that some interleukins could be used in the treatment of thyroid cancer and evaluation of curative effect. However, researches on the treatment of thyroid cancer by interleukin are limited to cell and animal researches. This may be because the mechanism of interleukins in thyroid cancer is not fully understood. Studies should further explore the mechanisms of interleukins in thyroid cancer in order to find more potential targets for the treatment of thyroid cancer. Besides, the interleukins, which has been proved to be effective in basic researches, should be studied in combination with other drugs.

## Conclusion

Basic researches have confirmed that interleukins have significant roles in thyroid cancer through different potential mechanisms. Few interleukins exhibit anti-tumor roles in thyroid cancer, while most show pro-tumor effects. Interleukins have promising prospect in clinical practice of thyroid cancer. They enhance the accuracy of diagnosis of thyroid cancer and provide novel treatments approaches. Besides, interleukins may also be used as biomarkers of surveillance for recurrence and serve as prognostic factors in thyroid cancer. However, there still some limitations on the clinical applications of interleukins in thyroid cancer. Firstly, only a few interleukins in thyroid cancer have been described, and potential mechanisms of other interleukins are not very clear. Secondly, there are some differences in the results of the clinical study on the utility of interleukins in thyroid cancer, which may be due to the different methods and different sample size. In addition, interleukins level in human is affected by many diseases, and to date there is no studies focusing on the accuracy and clinical value of interleukins in discriminating thyroid tumors combined with other diseases. Finally, the use of interleukins in the treatment of thyroid cancer is limited to cell and animal researches. However, what certain is that with future researches, interleukins will provide more help for clinicians and patients.

## Author Contributions

All authors listed have made a substantial, direct and intellectual contribution to the work, and approved it for publication.

## Conflict of Interest

The authors declare that the research was conducted in the absence of any commercial or financial relationships that could be construed as a potential conflict of interest.
